# Sex-biased transcription enhancement by a 5' tethered Gal4-MOF histone acetyltransferase fusion protein in *Drosophila*

**DOI:** 10.1186/1471-2199-11-80

**Published:** 2010-11-09

**Authors:** Anja H Schiemann, Fang Li, Vikki M Weake, Esther J Belikoff, Kent C Klemmer, Stanley A Moore, Maxwell J Scott

**Affiliations:** 1 Institute of Molecular BioSciences, Massey University, Private Bag 11222, Palmerston North, New Zealand; 2 Dept. of Biochemistry, University of Saskatchewan, 107 Wiggins Rd., Saskatoon, Saskatchewan S7N 5E5, Canada; 3 Stowers Institute for Medical Research, Kansas City, Missouri, USA; 4 Department of Genetics, North Carolina State University, Campus Box 7614, Raleigh NC 27695-7614, USA

## Abstract

**Background:**

In male *Drosophila melanogaster*, the male specific lethal (MSL) complex is somehow responsible for a two-fold increase in transcription of most X-linked genes, which are enriched for histone H4 acetylated at lysine 16 (H4K16ac). This acetylation requires MOF, a histone acetyltransferase that is a component of the MSL complex. MOF also associates with the non-specific lethal or NSL complex. The MSL complex is bound within active genes on the male X chromosome with a 3' bias. In contrast, the NSL complex is enriched at promoter regions of many autosomal and X-linked genes in both sexes. In this study we have investigated the role of MOF as a transcriptional activator.

**Results:**

MOF was fused to the DNA binding domain of Gal4 and targeted to the promoter region of UAS-reporter genes in *Drosophila*. We found that expression of a UAS-red fluorescent protein (DsRed) reporter gene was strongly induced by Gal4-MOF. However, DsRed RNA levels were about seven times higher in female than male larvae. Immunostaining of polytene chromosomes showed that Gal4-MOF co-localized with MSL1 to many sites on the X chromosome in male but not female nuclei. However, in female nuclei that express MSL2, Gal4-MOF co-localized with MSL1 to many sites on polytene chromosomes but DsRed expression was reduced. Mutation of conserved active site residues in MOF (Glu714 and Cys680) reduced HAT activity *in vitro *and UAS-DsRed activation in *Drosophila*. In the presence of Gal4-MOF, H4K16ac levels were enriched over UAS-*lacZ *and UAS-*arm-lacZ *reporter genes. The latter utilizes the constitutive promoter from the *arm *gene to drive *lacZ *expression. In contrast to the strong induction of UAS-DsRed expression, UAS-*arm-lacZ *expression increased by about 2-fold in both sexes.

**Conclusions:**

Targeting MOF to reporter genes led to transcription enhancement and acetylation of histone H4 at lysine 16. Histone acetyltransferase activity was required for the full transcriptional response. Incorporation of Gal4-MOF into the MSL complex in males led to a lower transcription enhancement of UAS-*DsRed *but not UAS-*arm-lacZ *genes. We discuss how association of Gal4-MOF with the MSL or NSL proteins could explain our results.

## Background

The male specific lethal (MSL) ribonucleoprotein complex is required for X chromosome dosage compensation in the fruit fly *Drosophila melanogaster *[1-3]. The MSL complex binds to most actively transcribed X-linked genes in males [4-6] and is responsible for a two-fold enhancement in gene transcription [7,8]. While there has been considerable progress in our understanding of the composition of the MSL complex [1,2] and the nature of the high affinity binding sites on the male X chromosome [9,10], less is known about the mechanism of transcription regulation. One protein component of the MSL complex that could play an important role in transcription enhancement is MOF, a member of the MYST family of histone acetyltransferase enzymes (HATs) [11]. In the presence of a nucleosomal substrate, purified MSL complex predominately monoacetylates histone H4 at lysine 16 (H4K16ac) [12,13]. The MSL complex has considerably less HAT activity when MOF has a single glycine to glutamic acid change in the acetyl-CoA binding motif (G691E, the *mof*^1 ^allele) [11]. In the presence of free histones recombinant MOF is less specific, preferentially acetylating the N-terminal tail of histone H4 but also acetylating the N-terminal tail of histone H3. The stringent H4K16 substrate specificity of the MSL complex requires a nucleosomal substrate and integration of MOF into the complex. Association of MOF with MSL1 and MSL3 appears to be particularly important for HAT specificity and activity [14]. The carboxyl terminal domain of MSL1, which acts as a scaffold for complex assembly, interacts with both MOF and MSL3 [14,15].

In addition to being a component of the MSL complex, MOF also associates with proteins that form the non-specific lethal or NSL complex [16]. Protein components of the NSL complex include NSL1, NSL2, NSL3, MCRS2 and MBD-R2. Genome-wide ChIP-chip studies of male cells found that MOF associates predominately with promoter regions of autosomal genes but has a bimodal distribution on male X-linked genes with peaks at both 5' and 3' ends [17]. In contrast, MSL1 and MSL3 show little binding to autosomal genes but are highly enriched across active X-linked genes, with a bias towards the 3' end [4,5,17]. H4K16ac is strongly enriched at the 5' region of autosomal genes that have high levels of bound MOF [17]. Kind *et al. *therefore suggest that MOF has a role in gene expression independent of the MSL complex. However, a subsequent study found little support that MOF was important for 5' H4K16ac of genes [18]. Early immunostaining studies of polytene chromosomes demonstrated that there is significant enrichment of H4K16ac on the male X chromosome [19]. Further, the MSL complex co-localized to the hundreds of sites on the X chromosome enriched for H4K16ac [20]. More recent ChIP-chip experiments found that nearly all actively transcribed X-linked genes are highly enriched for H4K16ac throughout the body of the gene but with a bias towards the middle and 3' end [18].

There is mounting evidence that genes enriched for H4K16ac have an altered chromatin structure resulting in elevated transcription. In the crystal structure of the nucleosome core particle, several hydrogen bonds and salt bridges were observed between the basic tail of histone H4 (K16 to N25) and acidic side chains of histones H2A and H2B of a neighbouring nucleosome core particle [21]. Acetylation of H4 N terminal lysine residues would reduce this association. Indeed, incorporation of H4K16ac into nucleosomal arrays abolished a salt-dependent compaction into 30 nm-like fibres [22]. *In vivo*, the MSL complex appears to counteract the effect of factors that promote compaction of the male X chromosome such as ISWI and HP1. For example, in homozygous *ISWI *or *Su(var)2-5 *(the gene encoding HP1) mutant male salivary gland nuclei the X chromosome has a bloated appearance, which required MSL complex function [23,24]. H4K16ac interferes with binding of ISWI to nucleosomal substrate *in vitro *and antagonizes *ISWI *function *in vivo *[23].

Transcription of nucleosomal templates *in vitro *is enhanced by incorporation of H4K16ac [12,25]. In yeast, the expression of a reporter gene was strongly stimulated by a Gal4-MOF fusion protein [12]. The reporter gene had multiple Gal4 binding sites in the promoter. In contrast, little transcription enhancement was obtained with a Gal4-MOF^1 ^fusion protein carrying a G691E mutation. These results were perhaps somewhat surprising as 80% of histone H4 is acetylated at K16 in yeast [26]. Indeed, it was not shown if the reporter was enriched for H4K16ac in the presence of Gal4-MOF [12]. While these studies support a role for H4K16ac in gene transcription, it has recently been reported that H4K16ac is more strongly associated with DNA replication timing than transcription in *Drosophila *cells [27]. Here we have targeted MOF to reporter gene promoters by fusing to the DNA binding domain of the yeast Gal4 protein. This would mimic the observed enrichment of MOF at gene promoters but not the 3' enrichment seen on X-linked genes in males. Our aim was to determine if tethering MOF to a promoter would stimulate transcription. If so, was transcription enhancement equal in *Drosophila *males and females?

## Results

### Gal4-MOF activates the UAS-RedStinger reporter gene expression more strongly in females than males

**Figure 1:**
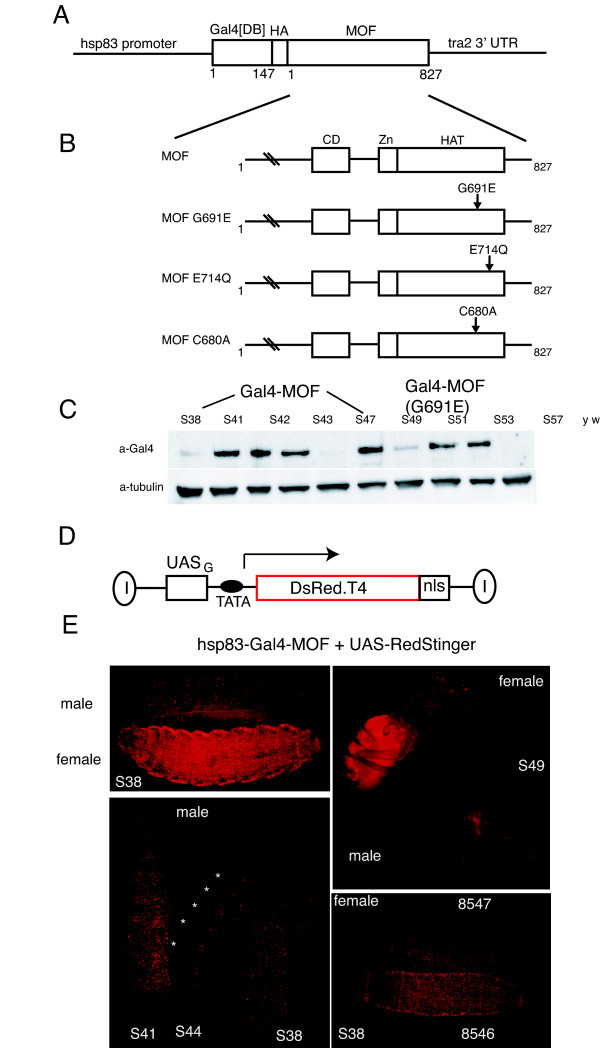
**Gal4-MOF activates expression of a UAS-RedStinger reporter gene**. (A, B). Schematic representation of the constructs used in this study. The DNA binding domain [DB] of yeast Gal4 (aa 1-147) was fused in frame with a HA epitope tag and either full length MOF or MOF mutant. Expression was controlled with the constitutive *hsp83 *promoter. The numbers in parentheses correspond to the amino acid numbers in full length MOF, the mutations analysed in MOF are indicated, mutated amino acids are numbered. CD: chromo-related domain; Zn: CCHC zinc finger; HAT: acetyl transferase domain. (C) Gal4-MOF protein expression in adult flies was confirmed by western blotting with an anti-Gal4 antibody. Blots were stripped and re-probed with an alpha-tubulin antibody as a loading control. Similar blots were performed for all lines used in this study. (D) Schematic representation of the UAS-RedStinger reporter [28]. Expression of the DsRed.T4 red fluorescent protein is under the control of a minimal promoter from the *hsp70 *gene and upstream binding sites for Gal4 (UAS). The reporter is flanked by *gypsy *insulator elements (I). The nuclear localization sequence (nls) from TRA is fused to the C terminus of DsRed.T4. (E) Gal4-MOF activates UAS-RedStinger expression more strongly in females than males in larvae (top left panel) and adults (top right panel). DsRed.T4 fluorescence is highest in males that express high levels of Gal4-MOF protein such as line S41 (bottom right panel). Reporter line UAS-RedStinger4 (FBst0008546) responds more strongly to Gal4-MOF than line UAS-RedStinger6 (FBst0008547) (bottom right panel).

The Gal4-MOF and Gal4-MOF[G691E] gene fusions previously used to control reporter gene expression in yeast [12] were cloned into a *Drosophila P-*element transformation vector that contained the constitutive *hsp83 *promoter (Figure 1A). A hemagluttinin (HA) epitope tag is present between the Gal4 DNA binding domain and MOF, which facilitates protein detection in cells. A control gene construct was made that contains only the DNA binding domain of Gal4 (Gal4[DB]). To confirm that any difference in reporter gene response between Gal4-MOF and Gal4-MOF[G691E] was due to loss of HAT activity, we made two additional active site MOF mutants, C680A and E714Q (Figure 1B, see below). Western blot analysis confirmed expression of the Gal4 fusion proteins (examples shown in Figure 1C). There was some variability in the level of Gal4 fusion protein expression between lines. Lines that had very high or very low levels of Gal4 fusion protein expression were not examined further.

At least three lines for each construct were selected for assays with a UAS-Red Stinger reporter (Figure 1D). This reporter encodes the fast maturing DsRed.T4 red fluorescent protein with a nuclear localization sequence (NLS) that is under the control of a minimal *hsp70 *promoter with upstream binding sites for Gal4 (UAS) [28]. In the presence of Gal4-MOF, strong activation of DsRed.T4-NLS expression was observed in female larvae (Figure 1E). Much less DsRed.T4-NLS expression was observed in male larvae. Similarly, female adults showed significantly higher levels of red fluorescence than adult males. DsRed.T4-NLS expression was particularly strong in the abdomen but expression was also detected in head and thorax. Subsequent experiments were performed using third instar larvae, as low levels of DsRed.T4-NLS expression could be detected since background fluorescence was low at this stage of development. The level of reporter gene activation correlated with the level of Gal4-MOF protein expression. This was most easily seen in male larvae, which have a lower level of reporter gene activation than female larvae. For example, more red fluorescence was observed with line S41, which makes moderate to high levels of Gal4-MOF, than either of the low expression lines S38 and S44 (Figure 1E). We noticed lateral clusters of cells that showed strong red fluorescence with all Gal4-MOF drivers, including lines that express low levels of protein. The location of the cell clusters and variable number of cells per cluster suggests that these are larval oenocytes [29]. One of the reporter lines, UAS-RedStinger4 [chromosome 2, FlyBase ID FBst0008546], responded more strongly to Gal4-MOF than the other available autosomal line UAS-RedStinger6 [chromosome 3, FlyBase ID FBst0008547]) (Figure 1E). Hence, for most experiments we used the UAS-RedStinger4 line. An X-linked reporter line, UAS-RedStinger3 [FlyBase ID FBst0008545], also responded more strongly to Gal4-MOF in females than males (Additional file 1, Figure S1).

### HAT activity is required for robust transcription enhancement by MOF

**Figure 2:**
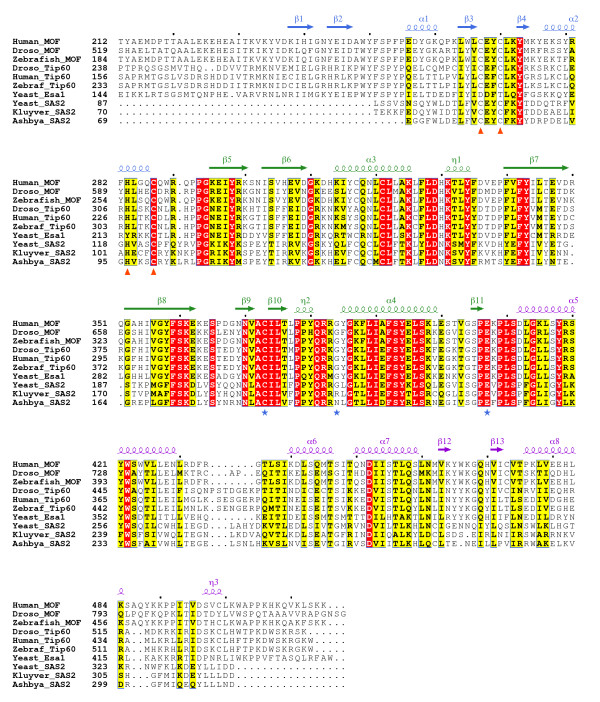
**Multiple sequence alignment of MOF and related MYST HAT domains**. Alignment calculated with T-Coffee (Notredame et al., 2000). Figure prepared with Espript (Gouet et al, 1999). N-terminal sequences including chromodomains were removed manually after the initial alignment was completed. Secondary structures derived from the atomic coordinates of the hMOF HAT domain (PDB ID 2PQ8; Temple, W. et al. Structural Genomics Consortium), and colored according to the domain representations depicted in Figure 3. Active site residues mutated in dMOF in this study are marked by blue stars. Zinc finger ligand residues are marked by orange triangles. Moderately conserved residues are boxed and have a yellow background. Strictly conserved residues are boxed with a red background. The sequences of the HAT domains are: Human_MOF (*Homo sapiens *MOF, gi14149875), Droso_MOF (*Drosophila melanogaster *MOF, gi3024151); Zebrafish_MOF (*Danio rerio *Hat1, gi 160774364); Droso_Tip60 (*Drosophila melanogaster *Tip60, gi18858193); Human Tip60 (*Homo sapiens *Tip60, gi 12652827); Zebraf_Tip60 (*Danio rerio *Tip60, gi225543380); Yeast Esa1 (*Saccaromyces cerevisiae *Esa1, gi3023717); Yeast_SAS2 (*Saccaromyces cerevisiae *SAS2, gi730713); Kluyver_SAS2 (*Kluyveromyces lactis *SAS2 gi50311675); Ashbya_SAS2 (*Ashbya gossypii *SAS2 homologue, gi45198739).

**Figure 3:**
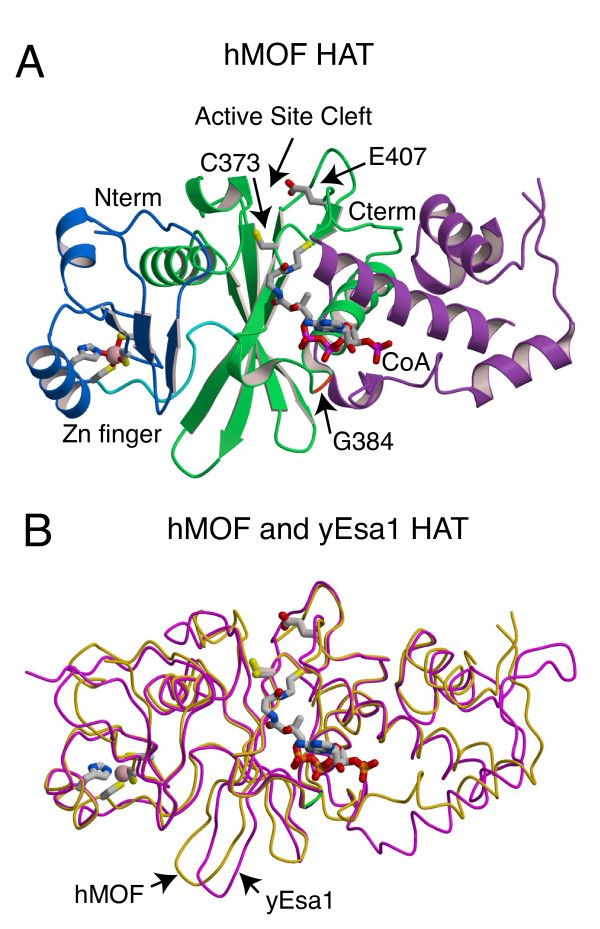
**Comparison of the structures of hMOF and yEsa1**. (A) Ribbon representation of hMOF acetyltransferase domain (hMOF PDB ID 2PQ8). Bound Coenzyme A is shown as a stick model, the atoms have standard colors. The Zn-finger domain of hMOF is colored blue, the central HAT domain green and the C-terminal domain is magenta. The amino acid side chains of the Zn-finger ligands (Cys 267, Cys 270, His 283, and Cys 287) are shown as a stick model. The Zn^2+ ^atom is shown as a pink sphere. The side chains of active site residues Cys 373 (dMOF Cys 680; yEsa1 Cys 304) and Glu 407 (dMOF Glu 714; yESA1 Glu 338) mutated in dMOF in this study are also shown as ball and stick models, and the active site cleft is labeled. The position corresponding to Gly 691 (*mof*^*1 *^mutation in *D. melanogaster *MOF) (hMOF Gly 384; yESA1 Gly 315) is shown in orange and labeled. (B) Structural superposition of the HAT domains of hMOF (yellow-orange) and yESA1 (magenta) (Esa1 PDB ID 1FY7). Drawn as in (A) except that the position of Gly 384 of hMOF is shown in green and the Coenzyme A moiety is shown only for hMOF (Esa1 CoA omitted for clarity). Superposition carried out using lsqkab of the CCP4 package. Figures drawn with Molscript [54] and Raster3D [55].

**Figure 4:**
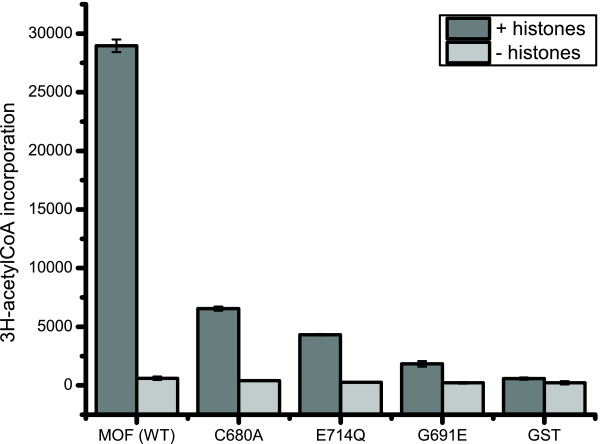
**HAT activity of MOF active site mutants**. Histone acetyl transferase (HAT) activity of recombinant MOF and MOF mutants with HeLa core histones as substrate. Incorporation [^3^H]acetyl-coenzyme A is shown in the presence or absence of histones.

We next asked if histone acetyltransferase (HAT) activity was essential for transcription activation by Gal4-MOF. We made the C680A and E714Q MOF point mutations based on the results of mechanistic studies identifying the homologous residues C304 and E338 as essential for HAT activity in yeast Esa1 [30,31]. Esa1 is a MYST HAT that is highly similar to MOF (Figure 2). The recent deposition of the protein coordinates of the crystal structure of human MOF (hMOF) allowed us to compare the hMOF and Esa1 three dimensional structures (Figure 3). Overall, hMOF and Esa1 share a high degree of structural similarity. In particular the active site glutamate and cysteine residues occupy almost identical positions. The hMOF HAT domain structure is also highly similar to the three dimensional structures of other human MYST family HATs, hTip60 and MOZ (not shown). Hence, we predicted that the MOF mutants containing C680A or E714Q point mutations should have greatly reduced HAT activity. To test this prediction, HAT assays were performed with purified recombinant MOF wild type and mutant proteins and HeLa core histones. Consistent with previously published studies [12,13], we found that recombinant MOF containing the chromo and MYST HAT domains (aa 370-827) catalysed acetylation of histone substrate but MOF[G691E] was at least ten times less active (Figure 4). MOF[C680A] and MOF[E714Q] were about 5 to 8 times less active than MOF (Figure 4). This supports the prediction that the active site glutamate and cysteine residues would be important for catalysis.

**Figure 5:**
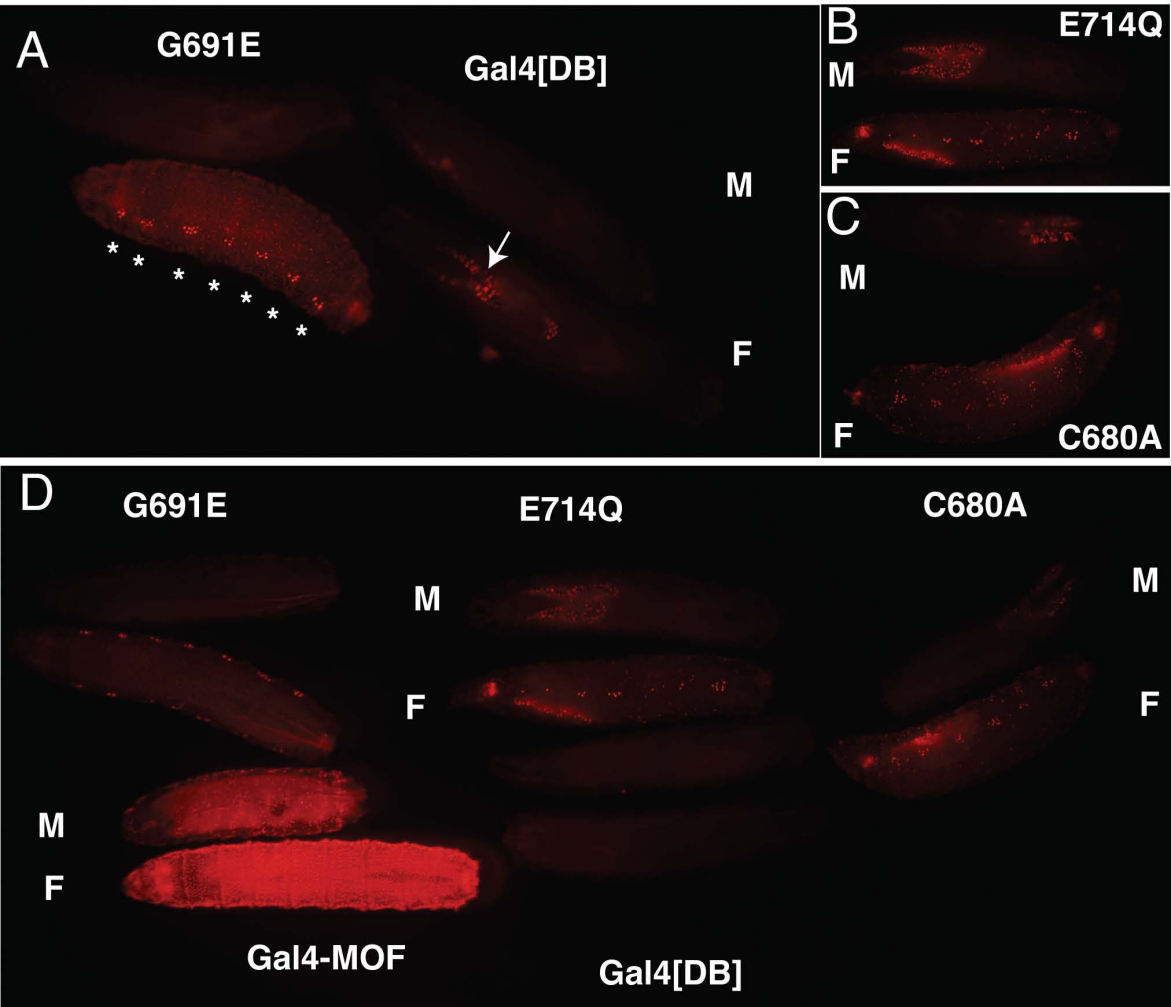
**HAT activity is required for robust transcription activation by Gal4-MOF**. Larvae carry the UAS-RedStinger4 reporter and a *hsp83*-Gal4-MOF mutant transgene. (A) hsp83-Gal4-MOF[G691E] line S53 and *hsp83*-Gal4[DB] line S74, (B) hsp-Gal4-MOF[E714Q] line S67, (C) *hsp83*-Gal4-MOF[C680A] line S72, (D) Comparison of larvae that express Gal4-MOF (line S49) with larvae that express a Gal4-MOF mutant (lines S53, S67, S72) or Gal4(DB) control (line S74). Reporter gene expression is higher in female (F) than male larvae (M). Strong expression is observed in oenocytes (asterisk, panel A) in female larvae that express a Gal4-MOF HAT mutant. All larvae express the DsRed.T4 reporter in salivary glands, including Gal4[DB] control (e.g. arrow, panel A), but this is not seen in some of the larvae shown because of orientation and focal plane.

In the presence of Gal4-MOF[G691E] protein, DsRed.T4-NLS expression was higher in female than male larvae (Figure 5A). The level of red fluorescence in male larvae was similar to the Gal4[DB] control. In female larvae, red fluorescence was particularly noticeable in larval oenocytes and salivary glands. However, DsRed.T4-NLS expression in salivary gland was also detected with the Gal4[DB] control, as previously reported [32]. That is, since the DNA binding domain of Gal4 is sufficient to activate expression in salivary glands, all Gal4-MOF active site mutant fusion proteins showed DsRed expression in salivary gland. The results shown are typical for the three Gal4-MOF[G691E] lines that were examined. Similarly, low but readily detectable levels of DsRed.T4-NLS expression were observed in female larvae with either Gal4-MOF[E714Q] (Figure 5B) or Gal4-MOF[C680A] proteins (Figure 5C). In male larvae, DsRed.T4-NLS expression was similar to the Gal4[DB] control. The level of red fluorescence in female larvae that express a Gal4-MOF active site mutant was much less than female larvae that express Gal4-MOF (Figure 5D). As similar results were obtained with all three HAT mutants we conclude that MOF HAT activity is required for robust reporter gene transcription activation by Gal4-MOF. However, HAT activity does not appear to be essential for transcription activation by MOF.

**Figure 6:**
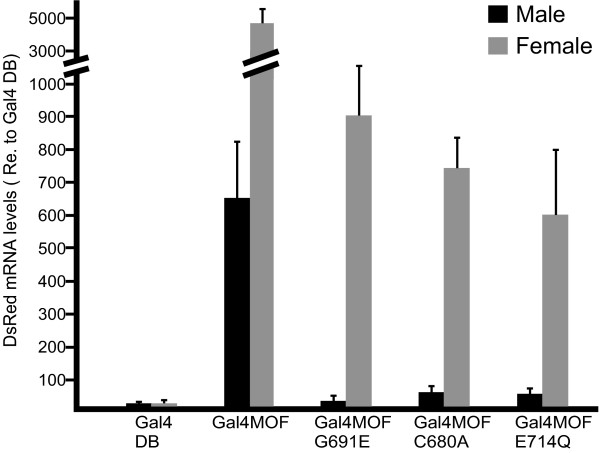
**DsRed.T4-NLS RNA levels in larvae that express Gal4-MOF fusion proteins**. Quantitative real time RT-PCR was used to determine the levels of DsRed RNA in samples from sex-sorted larvae. *pka *RNA levels were used for normalization. Three biologically independent experiments were performed and each sample was assayed in triplicate. Mean and standard deviation are shown. The DsRed RNA levels in the Gal4-MOF and Gal4-MOF mutant lines are plotted relative to the Gal4[DB] control.

To more accurately quantify reporter gene expression levels, we performed real-time quantitative PCR with RNA isolated from larvae that contain the UAS-Red Stinger reporter and express a Gal-MOF protein or Gal4[DB] control. Samples from three independent experiments were analyzed in triplicate. *pka *RNA levels were used for normalization of DsRed RNA levels. The results are shown in Figure 6. The increase in DsRed RNA is shown relative to the RNA level measured in the Gal4[DB] control. The results are consistent with the fluorescence microscopy observations. A large increase in DsRed RNA is seen in female larvae that express Gal4-MOF. Gal4-MOF males make about 7 times less DsRed RNA. Female larvae that express one of the active site mutants ([G691E], [E714Q] or [C680A]) contain significant levels of DsRed RNA, comparable to males that express Gal4-MOF. In contrast, much less DsRed RNA was detected in male larvae that express Gal4-MOF [G691E], [E714Q] or [C680A]. The level of induction of DsRed RNA in female larvae by the MOF active site mutants suggests that histone acetylase activity is not essential for transcription activation. However, it is possible that the transcription activation is due to residual acetyltransferase activity (Figure 4). Alternatively, transcription activation could be due to an NSL protein(s) that associates with the Gal4-MOF mutant proteins and is thus recruited to the promoter of the reporter gene (see Discussion below).

### Gal4-MOF is incorporated into the MSL complex

**Figure 7:**
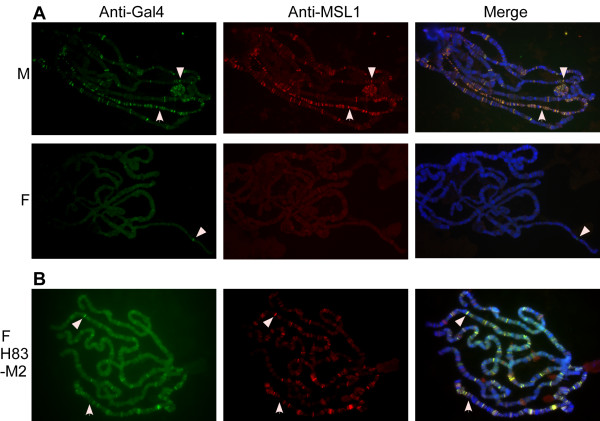
**Gal4-MOF co-localizes with MSL1 on polytene chromosomes**. (A) Male (top) and female (bottom) larvae salivary gland nuclei that express Gal4-MOF and carry a UAS-*lacZ *transgene were stained with anti-Gal4 (green), anti-MSL1 (red) and counterstained with DAPI (blue). Gal4-MOF and MSL1 co-localize to many sites on the X chromosome (arrowhead) and autosomes in male nuclei (yellow, right panel). In female nuclei, Gal4-MOF binding is reduced to a few sites, the brightest of which corresponds to the location of the UAS-*lacZ *transgene (arrow). (B) Gal4-MOF co-localizes with MSL1 to many chromosomal sites in female nuclei that constitutively express MSL2.

We considered several possible explanations for why Gal4-MOF more strongly activated the UAS-RedStinger reporter in female larvae. One possibility was that if Gal4-MOF was incorporated into the MSL complex in males, then less Gal4-MOF would be available to bind to the reporter gene. To evaluate this possibility we examined polytene chromosomes of third instar larvae salivary glands that express Gal4-MOF and carry a UAS-*lacZ *gene. In female nuclei, Gal4-MOF bound to a few sites on the X chromosomes and autosomes (Figure 7A). In general binding was at a low level, although strong binding was consistently seen at the site of the UAS*-lacZ *transgene (Figure 7A, arrow). In male nuclei, Gal4-MOF bound to hundreds of sites on the male X chromosome and to many sites on the autosomes. Binding was generally stronger than seen in female nuclei. MSL1 co-localized with Gal4-MOF to sites on the X chromosome and autosomes in male nuclei. In contrast, MSL1 did not co-localize with Gal4-MOF to the site of the UAS-*lacZ *transgene in female nuclei. In wild type male nuclei there is little binding of MSL1 to the autosomes [33]. This suggests that Gal4-MOF has been incorporated into the MSL complex but has somehow altered the normal binding profile of the complex. Possibly the fusion of the DNA binding domain of Gal4 has altered the normal function of MOF. If so, Gal4-MOF may not be able to functionally replace MOF in the MSL complex. Thus, we next asked if Gal4-MOF could rescue *mof*^*1 *^mutant males, which die at third instar or prepupal stage [11]. We found that expression of Gal4-MOF (line S49) did not rescue males from the lethal effects of the *mof*^*1 *^mutation (Table 1). Similar results were obtained with a different Gal4-MOF line (data not shown). Thus it appears that, although the fusion of the DNA binding domain of Gal4 to the amino end of MOF has not disrupted HAT activity (see below) or incorporation into the MSL complex, the MSL complex containing Gal4-MOF is not fully functional. In their recent study, Becker and colleagues reported that *mof*^*1 *^males were rescued by expression of a Gal4-MOF fusion protein driven by the endogenous *mof *promoter [34]. Thus an alternative explanation for our results is that the *hsp83 *promoter does not provide the required expression profile of MOF fusion protein to rescue *mof*^*1 *^males.

**Table 1:** Gal4-MOF does not rescue males from the lethal effects of the *mof*^*1 *^mutation^a^

**mof**^**1**^**/Y; S49/+ male**	FM7/Y; S49/+ male	**mof**^**1**^**/+; S49/+ female**	FM7/+; S49/+ female
0	117	156	167

^a^Full genotype of cross: *w cv mof*^*1*^; FM7, y^31d ^sc w^a ^B females crossed with *w*^1 ^*y*^1^; S49 {*w*+, H83-Gal4-MOF}/TM3, *Ser y*+ males. For clarity, the number of flies that inherited the TM3, Ser balancer chromosome are not shown.

The MSL complex does not assemble in females because translation of *msl2 *RNA is repressed by SXL [35]. However, constitutive expression of MSL2 from a transgene causes the MSL complex to assemble in females [36]. If the difference in the chromosome-binding pattern between male and female nuclei is because Gal4-MOF is incorporated into the MSL complex in male nuclei, then constitutive expression of MSL2 in females should lead to an altered distribution of Gal4-MOF. As predicted, in female nuclei that carry the *hsp83-msl2*, *hsp83*-Gal4-MOF and UAS-*lacZ *transgenes, we observed strong binding of Gal4-MOF to many sites on the X chromosomes and autosomes (Figure 7B). The binding of Gal4-MOF was stronger than seen in female nuclei that don't express MSL2 (Figure 7A). Further, MSL1 co-localized with Gal4-MOF, confirming incorporation of Gal4-MOF into the MSL complex. The enrichment of the MSL complex on X chromosomes was less than typically observed in female nuclei that express MSL2 [37], which is consistent with the interpretation that Gal4-MOF has interfered with the normal chromatin binding pattern of the MSL complex.

**Figure 8:**
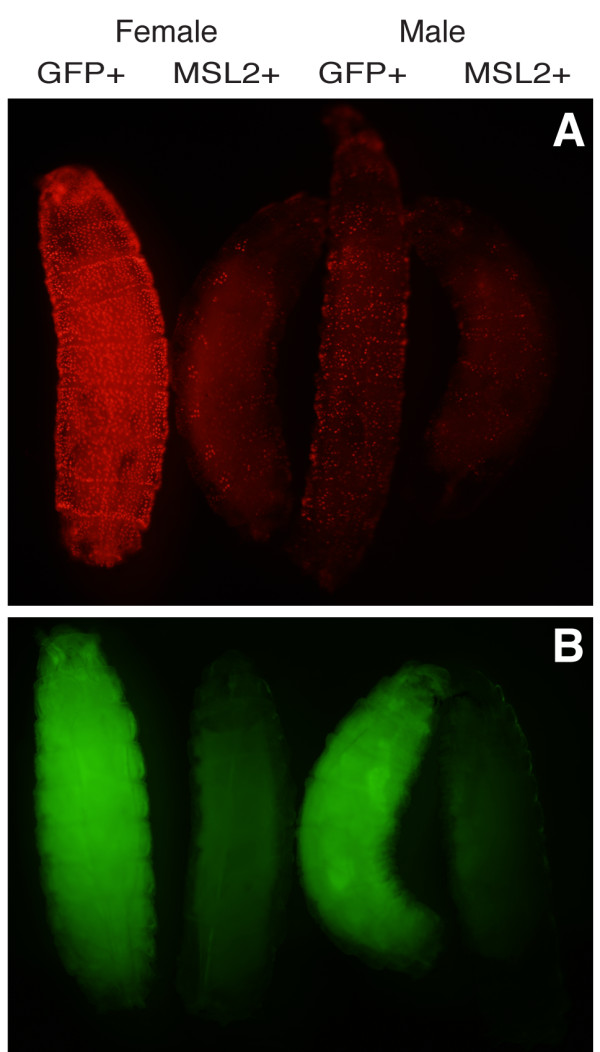
**Gal4-MOF dependant reporter gene expression is reduced in female larvae that express MSL2**. Recombinant *hsp83*-Gal4-MOF, UAS-RedStinger females were crossed with *hsp83*-MSL2/PUb-EGFP males and the larval offspring were observed with green (A) or blue (B) filter sets. The GFP minus larvae constitutively express MSL2. Female larvae on left and male larvae on right of the panels.

We next examined DsRed fluorescence in female larvae that constitutively express MSL2 and Gal4-MOF and carry the UAS-RedStinger reporter gene. We observed a dramatic decrease in DsRed.T4 fluorescence in female larvae that express MSL2 (Figure 8A). The strongest fluorescence was seen in oenocytes, as also seen in male larvae that express Gal4-MOF. Interestingly, there was also a small but consistent decrease in DsRed.T4 fluorescence in male larvae that express MSL2 compared to normal males. These results suggest that if more Gal4-MOF protein is driven into the MSL complex then there is less activation of the UAS-RedStinger reporter gene.

**Figure 9:**
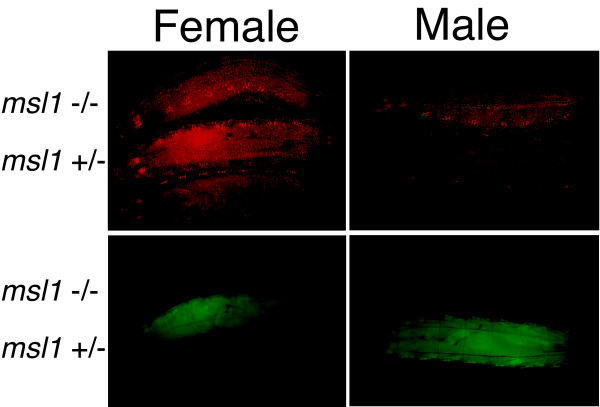
**Gal4-MOF dependant reporter gene expression is increased in *msl1 *mutant male larvae**. All larvae carry a *hsp83*-Gal4-MOF driver and UAS-RedStinger reporter gene. *msl1*^*L60 *^homozygous males and females were distinguished from heterozygous siblings by using a GFP marker.

If low DsRed.T4 expression in males is because Gal4-MOF is incorporated into the MSL complex then reporter gene expression should increase in *msl1 *mutant males. Alternatively, since MOF requires MSL1 for robust histone acetyltransferase activity *in vitro *[14], reporter gene expression driven by Gal4-MOF could decrease in *msl1 *mutant males and females. Homozygous and heterozygous *msl1*^*L60 *^larvae were distinguished using a constitutively expressed GFP marker for the second chromosome that had a normal copy of the *msl1 *gene. For these experiments it was necessary to use the third chromosome-linked UAS-RedStinger6 reporter, which shows a significant response to Gal4-MOF but the response is less strong than the UAS-RedStinger4 line used previously. We observed higher DsRed.T4 fluorescence in homozygous *msl1*^*L60 *^male larvae compared to their heterozygous brothers (Figure 9A). In female larvae, DsRed.T4 expression was similar in homozygous and heterozygous *msl1*^*L60 *^mutants. These results show that the lower reporter gene response in males is because Gal4-MOF is incorporated into the MSL complex. Further, it does not appear that MSL1 is essential for transcription activation by Gal4-MOF.

### Gal4-MOF elevates expression of a UAS-*arm-lacZ *reporter gene

The UAS-RedStinger reporter gene is an artificial construct consisting of five Gal4 DNA binding sites and a minimal promoter from the autosomal *hsp70 *gene. In the absence of activator, background DsRed.T4 expression is negligible. To examine the effect of Gal4-MOF on a promoter from an X-linked gene, we inserted twelve copies of the Gal4 binding site upstream of the *armadillo *promoter in the *arm-lacZ *reporter. We called this reporter 3 × UAS*-arm-lacZ*. We previously used the *arm-lacZ *reporter to examine the effect of MSL complex binding on gene expression [8,38]. We found that insertion of a strong MSL complex binding site upstream of the *arm *promoter led to a two-fold increase in *lacZ *expression in males [8]. In contrast to the robust transcription activation of the UAS-RedStinger reporter, there was only a modest increase in *lacZ *expression in hemisected adults in the presence of Gal4-MOF (Table 2). Further, the increase in *lacZ *expression was similar in males and females, in contrast to the female-biased RedStinger expression seen in larvae. However, the increase in *lacZ *expression was significant in both sexes (ANOVA, P < 0.01). We also measured β-galactosidase activities in larvae and found only a modest increase in 3 × UAS*-arm-lacZ *expression in males (1.67 +/- 0.14) and females (2.29 +/- 0.47) in the presence of Gal4-MOF.

**Table 2:** Transcription regulation of a 3 × UAS*-arm-lacZ *reporter by Gal4-MOF fusion proteins in hemisected adults

Construct	Line	**n**^**a**^	**Mean male relative β-galactosidase activities**^**b**^	**Mean female relative β-galactosidase activities**^**b**^
*arm*-Gal4	1561	3	1.04 ± 0.06^c^	1.1 ± 0.0003
*hsp83*-Gal4[DB]	S74	3	1.057 ± 0.041	0.96 ± 0.031
	S75	3	0.95 ± 0.06	0.95 ± 0.03
*hsp83*-Gal4-MOF	S41	3	2.17 ± 0.12	1.67 ± 0.08
	S46	3	2.47 ± 0.3	2.05 ± 0.21
*hsp83*-Gal4-MOF(G691E)	S52	3	1.78 ± 0.11	1.54 ± 0.05
	S53	3	1.56 ± 0.09	1.30 ± 0.03
*hsp83*-Gal4-MOF(E714Q)	S64	3	1.70 ± 0.03	1.49 ± 0.12
	S65	3	1.32 ± 0.27	1.04 ± 0.01
*hsp83*-Gal4-MOF(C680A)	S72	3	1.45 ± 0.22	1.60 ± 0.15
	S73	3	1.32 ± 0.13	1.27 ± 0.11

^a^Number of independent experiments

^b^Relative to β-galactosidase activity of control 3 × UAS-*arm-lacZ *hemisected adults

^c^Mean +/- standard deviation. Activity in 100 mOD min^-1 ^mg protein

In hemisected adults, the β-galactosidase activity in the UAS-*arm-lacZ *strain was well above the background activity in the parental *y w *strain (not shown), as anticipated from previous studies [8,38]. There was no significant elevation in *lacZ *expression with either Gal4[DB] or full length Gal4 (Table 2). The lack of stimulation by full length Gal4 was perhaps not surprising as the binding sites are greater than 650 bp upstream from the first transcription start site of the *arm *gene. In yeast [39] and *Drosophila *[40] the level of transcription stimulation by Gal4 decreases with increased distance between binding sites and the promoter. Surprisingly, in the presence of either Gal4-MOF(G691E), Gal4-MOF(C680A) or Gal4-MOF(E714Q), *lacZ *expression was also significantly greater than the respective *arm-lacZ *controls (ANOVA, P < 0.01 for both sexes). There was, however, some line-to-line variation in the degree of elevation of *lacZ *expression (e.g *hsp83*-Gal4-MOF(E714Q) lines S64 and S65). However, the elevation in *lacZ *expression with any of the MOF mutants was consistently less than observed with Gal4-MOF. In males the elevation in *lacZ *expression by Gal4-MOF was significantly higher than the increase in *lacZ *expression with any of the Gal4-MOF mutants (ANOVA, P < 0.05). We conclude that recruitment of Gal4-MOF to the *arm-lacZ *reporter leads to a small but significant increase in gene expression, which at least in part, is due to MOF HAT activity.

**Figure 10:**
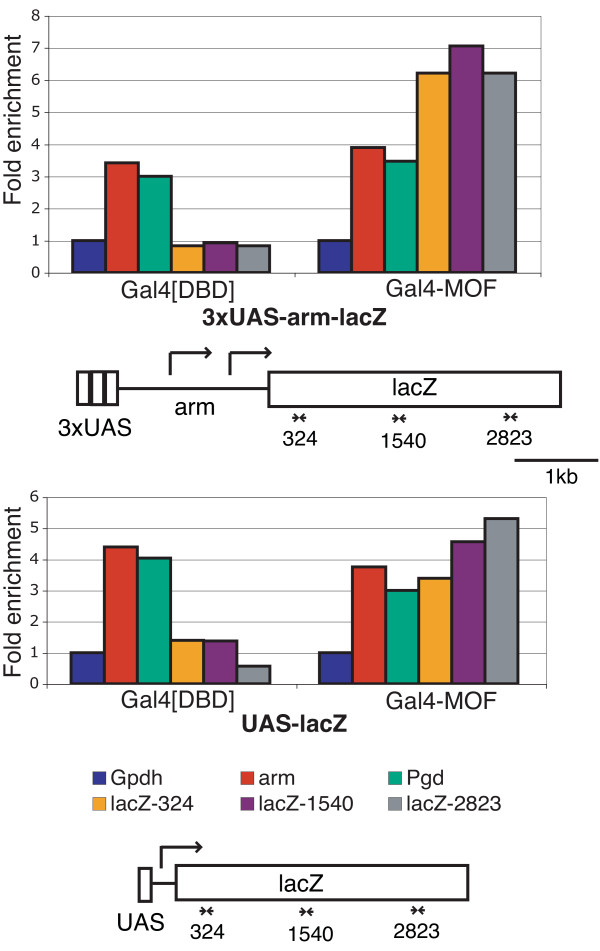
**H4K16ac is enriched at 3 × UAS*-arm-lacZ *and UAS*-lacZ *reporter genes in the presence of Gal4-MOF**. Flies carrying the *hsp83*-Gal4[DB] or *hsp83*-Gal4-MOF transgenes were crossed to 3 × UAS*-arm-lacZ *lines or UAS-*lacZ*. Chromatin from third instar larval offspring was immunoprecipitated with antibody against H4K16ac. The fold enrichment of immunoprecipitated DNA relative to input DNA is shown. Fold enrichment is normalized to the autosomal *Gpdh*, which is set to 1. A 3-4 fold enrichment is observed for transcribed regions of the control X-linked genes *Pgd *and *arm*. Three primer sets were used to amplify different regions within the *lacZ *gene. A 3 to 7-fold enrichment is observed for the *lacZ *gene in the lines expressing Gal4-MOF compared to lines expressing Gal4[DB]. A schematic illustration of the 3 × UAS*-arm-lacZ *and UAS-*lacZ *reporter genes with relative location of primer pairs used to amplify *lacZ *sequences is shown below each graph. Arrows indicate the transcription start sites.

In the 3 × UAS-*arm-lacZ *reporter, the Gal4 binding sites are about 1.8 kb upstream of the *lacZ *gene. We next asked if binding of Gal4-MOF led to an increase in H4K16ac over the body of the *lacZ *gene. Chromatin immunoprecipitation was performed using an antibody that recognises H4K16ac. We found that there was an enrichment for H4K16ac across the *lacZ *gene in nuclei from third instar larvae of mixed sex (Figure 10). There was no enrichment of H4K16ac in the *lacZ *gene in larvae that express the Gal4[DB] control protein. Thus binding of Gal4-MOF upstream of the promoter does lead to an increase in H4K16ac levels across the 3 × UAS*-arm-lacZ *reporter gene. We also examined larvae that express Gal4-MOF and have a *lacZ *reporter with a minimal promoter and Gal4 binding sites immediately upstream of the promoter (UAS-*lacZ*). As the Gal4-MOF binding sites are very close to the start of transcription we predicted that the *lacZ *gene would be enriched for H4K16ac. Indeed, we found an enrichment for H4K16ac across the *lacZ *gene in larvae of mixed sex (Figure 10). Similar enrichments in H4K16ac were observed in independent ChIP experiments with the *lacZ *reporter genes (Additional file 2, Figure S2).

## Discussion

In this study we asked if targeting MOF to a reporter gene would lead to an enrichment of H4K16ac across the gene and therefore result in transcription enhancement in *Drosophila*. We found that H4K16ac was increased over a reporter gene driven either by a minimal promoter with low basal activity or by a more generally active promoter from the X-linked *armadillo *gene. The increase in H4K16ac was significant because robust transcription enhancement by MOF required full HAT activity. However, the answer to the question of whether or not targeting MOF to a gene would enhance transcription was more complex than initially supposed. The degree of transcription enhancement depended upon the sex, tissue, reporter gene location and promoter. Interestingly, targeting MOF to the *arm-lacZ *reporter gene led to an approximately two-fold increase in gene expression. We had previously observed a two-fold increase in *lacZ *expression in males that carried the *arm-lacZ *reporter with an upstream MSL complex high affinity binding site [8]. Our results are consistent with the suggestion that the increase in H4K16ac over X-linked genes in males plays an important role in doubling gene expression [3].

We found that the human MOF and yeast Esa1 three dimensional structures were very similar. Importantly, the active site glutamate and cysteine residues occupied almost identical positions. In the proposed ping-pong mechanism for Esa1 catalysis, C304 acts to nucleophilically displace the acetyl group from acetylCoA [30,31]. Then E338 deprotonates the substrate lysine amino group to nucleophilically attack the acetyl-C304 covalent adduct, generating acetyl-lysine as the final product. The Esa1 mutants E338Q and C304A had essentially no HAT activity *in vitro*. While another *in vitro *study suggested that C304 did not play an essential role in the Esa1 mechanism [41], an *in vivo *study supports the earlier conclusion that C304 and E338 are essential for HAT activity in Esa1 [42]. We found that the highly conserved active site cysteine and glutamate amino acids were important for MOF catalytic activity *in vitro *and for UAS reporter gene activation in *Drosophila*.

We observed a particularly strong reporter gene response to Gal4-MOF in larval oenocytes. This was most easily seen in male larvae that otherwise responded weakly to the activator and in female larvae that expressed an active site mutant form of MOF. Presumably there is a tissue-specific factor in these cells that enhances the transcription stimulation by MOF. Oenocytes are highly specialized cells that regulate lipid metabolism in *Drosophila *[43], so it is not unlikely that such tissue-specific factors may exist.

Gal4-MOF more strongly activated expression of the UAS-RedStinger reporter gene in females than males. There are several lines of evidence that this is because Gal4-MOF is incorporated into the MSL complex in males. Firstly, Gal4-MOF co-localized with MSL1 in male salivary gland polytene chromosomes to sites on the X chromosome and autosomes. In female nuclei the strongest binding site corresponded to the location of the *UAS-lacZ *reporter gene. Gal4-MOF co-localized with MSL1 in female nuclei that constitutively express MSL2, and Gal4-MOF only weakly activated the UAS-RedStinger reporter in these females. Lastly, UAS-RedStinger was activated more strongly by Gal4-MOF in males that were unable to assemble the MSL complex due to a mutation in the *msl1 *gene.

There are three possible explanations for why incorporation of Gal4-MOF into the MSL complex in males would reduce activation of the UAS-RedStinger reporter gene. Firstly, because Gal4-MOF is sequestered into the MSL complex in males, there is less free protein available to bind to the autosomal UAS-reporter gene and enhance transcription. A similar model was proposed by Birchler and colleagues to explain a decrease in expression of autosomal *mini-white *and *yellow *transgenes in females that constitutively express MSL2 [44]. A second explanation is that the MSL complex somehow largely represses the transcription enhancement of the UAS-RedStinger reporter gene that would be expected from the increased histone acetylation by MOF. This is similar to the proposed role of the MSL complex in the "inverse dosage effect" model for X chromosome dosage compensation [45]. In this model the MSL complex sequesters MOF to the X chromosome reducing the inverse dosage effect on the autosomes by decreasing histone acetylation. Further the MSL complex is proposed to inhibit any transcription elevation of genes on the X chromosome due to increased level of H4K16ac. After submission of this manuscript, Becker and colleagues published their study on transcription regulation of reporter genes by a Gal4-MOF fusion protein [34]. Consistent with our findings, they found that expression of a UAS-lacZ reporter gene was higher in females than males and that the gene was enriched for H4K16ac. The authors favored a model that the MSL complex inhibits the transcription elevation due to increased H4K16ac such that the net effect is a two-fold increase in reporter gene expression. In contrast to our study, Prestel *et al. *(2010) did not test if catalytically inactive versions of MOF increased reporter gene expression, so it is unclear what fraction of transcription elevation by Gal4-MOF was due to MOF histone acetyltransferase activity. A third explanation for why reporter gene expression was higher in females is that more of the Gal4-MOF fusion protein is available to be incorporated into the NSL complex. In a recent study, Akhtar and colleagues found that the NSL complex is a potent regulator of gene expression, upregulating the expression of most target genes [46]. The authors provide several lines of evidence that the NSL complex members and MOF act synergistically to regulate gene expression. For example, a Gal4-NSL3 fusion protein is a potent activator of expression of a UAS-luciferase reporter gene but full activation required MOF and other NSL components. Thus if more NSL complex is assembled at the promoter of the UAS-RedStinger reporter gene in females than males, this could explain the higher expression observed in females. Indeed, Prestel et al (2010) showed that Gal4-MOF is incorporated into the NSL complex in female cells. They also found that the NSL complex regulates the expression of many genes in *Drosophila*. The recruitment of NSL proteins such as NSL3 by Gal4-MOF active site mutant proteins to the promoter region of the UAS-RedStinger reporter gene could explain why we observed a significant increase in DsRed expression in females. Presumably, in males the incorporation of Gal4-MOF mutant protein into the MSL complex and reduced HAT activity led to a very small increase in reporter gene expression compared to the Gal4[DB] control.

Interestingly, a recent study found that in the human NSL complex, MOF has relaxed substrate specificity relative to the MSL complex and acetylates histone H4 at lysines 5 and 8 in addition to lysine 16 [47]. During activation of transcription of the human IFNβ gene, acetylation of histone H4 at K8 leads to recruitment of the SWI/SNF chromatin remodelling complex [48]. It will be interesting to determine if the broader specificity of MOF when incorporated into the NSL complex in part explains why the NSL complex is a potent transcription regulator. Further, the "repressive" effect of the MSL complex on transcription activation by MOF may simply reflect the high specificity of MOF for H4K16 when part of the MSL complex.

In contrast to the sex-bias observed with the UAS-RedStinger reporter gene, both males and females showed only a very modest increase in 3 × UAS-*arm-lacZ *expression. One difference between the reporter gene constructs is that the baseline expression of *arm-LacZ *driven by the active *armadillo *promoter is much higher than the UAS-RedStinger reporter (or UAS-*lacZ*), which has a minimal promoter. It has recently been reported that knockdown of *mof *expression in *Drosophila *S2 had a greater effect on those autosomal genes that were expressed at low levels [49]. Further, reducing *mof *RNA levels had only a modest effect on autosomal gene expression. This might explain why only a small transcription enhancement was observed with the 3 × UAS-*arm-lacZ *reporter. Presumably, the NSL complex is recruited by Gal4-MOF to both of the reporter genes. However, the NSL complex is bound to the promoter region of the *arm *gene at its normal location on the X chromosome [34,46]. Thus, if the NSL complex is bound to the *arm *promoter in the 3 × UAS-*arm-lacZ *autosomal transgene, recruitment of additional NSL complex by Gal4-MOF may have little stimulatory effect on *lacZ *expression. Alternatively, NSL complex may be recruited to the 3 × UAS-*arm-lacZ *but may have little effect on transcription as the Gal4 binding sites are further upstream from the transcription start site than for the UAS-RedStinger reporter. In this regard, we observed that full length Gal4 did not stimulate 3 × UAS-*arm-lacZ *expression but Gal4 is a potent activator of UAS-*lacZ*. Surprisingly, expression of the 3 × UAS-*arm-lacZ *reporter gene was increased significantly by all three Gal4-MOF active site mutant proteins. This could be due to residual HAT activity of the mutant proteins (Figure 4). Alternatively, as discussed above, the transcription elevation of the reporter gene could be due, in part, to recruitment of other proteins to the *arm *promoter by the Gal4-MOF mutant proteins.

## Conclusions

In this study we have shown that targeting the histone acetyltransferase MOF to reporter genes via the DNA binding domain of Gal4 led to transcription enhancement and acetylation of histone H4 at lysine 16. Highly conserved active site residues Cys680 and Glu714 were important for MOF catalytic activity *in vitro *and for UAS-reporter gene activation in *Drosophila*. Gal4-MOF strongly induced expression of a UAS-DsRed reporter gene, particularly in females. In males, Gal4-MOF was incorporated into the male specific lethal (MSL) complex. The lower UAS-DsRed response in males could be because there is less Gal4-MOF protein available to bind to the reporter gene or the MSL proteins inhibit the transcription activation by MOF. Alternatively, it could be that the reporter gene response is higher in females as more Gal4-MOF protein is available to recruit the non-specific lethal (NSL) complex proteins to the promoter of the reporter gene. In contrast, Gal4-MOF only modestly increased expression of a 3 × UAS-*arm-lacZ *reporter driven by a constitutive promoter. This could be because of a higher basal activity of the reporter, greater distance between the Gal4 binding sites and the transcription start site or because the *arm *promoter can independently recruit the NSL complex.

## Methods

### Construction of plasmids

pAS2-MOF and pAS2-MOF G691E plasmids [12] were used as a template for PCR. The Gal4-MOF and Gal4-MOF G691E constructs were made by PCR using primers AS7 5'-TTCGGTACCGAAGCAAGCCTCCTG-3' and AS8 5'-TTCGGTACCCCCGGGCTAGCCGGAATTACCCGG-3". The PCR products were digested with *Asp*718 and cloned into pCaSpeR-h83. The Gal4-MOF plasmid served as a template to make the Gal4-MOF[E714Q] and Gal4-MOF[C680A] constructs by PCR using primers carrying the point mutations. The Gal4 DB construct was made by PCR using AS7 5'-TTCGGTACCGAAGCAAGCCTCCTG-3' and AS9 5'-ATAAAGAATGCGGCCGCCTACGGCGATACAGTCAAC-3". The PCR product was digested with *Kpn*I and *Not*I and cloned into pCaSpeR-h83. The UAS Gal4 binding site was excised from plasmid pBS-2N17mer by digestion with *Not*I and inserted into pRHO7 [8] to create p3 × UAS-*arm-lacZ*. For expression of MOF in *E. coli*, a fragment of the Drosophila *mof *open reading frame encoding amino acids 370 to 827 was inserted into the GST expression vector pGEX-6P3. This fragment contains the intact chromodomain and the MYST HAT domain. Active site mutants were made with the Stratagene quick change kit and verified by DNA sequencing. Primer sequences are available upon request.

### Recombinant MOF purification and HAT assays

*E. coli *cell pellets were resuspended in cleavage buffer (50 mM Tris-HCl [pH 8.0], 150 mM NaCl, 1 mM EDTA), protease inhibitors were added and then treated with 1 mg/mL hen egg white lysozyme (Sigma), 2.5 mg of DNaseI (10 mg/mL), and 50 U Benzonase (Invitrogen) per liter of cells at 4°C for 25 min. GST-fusion proteins were purified using Glutathione-Sepharose (GS) affinity chromatography with an ÄKTA FPLC and a HR 16/5 column (GE Life Science) packed with 10 - 15 mL of Glutathione SepharoseTM 4B (GE Life Science). Eluted protein was concentrated using an Ultracel regenerated cellulose Amicon^® ^Ultra-15 centrifugal filter device. HAT assays were performed in triplicate with each sample with [^3^H]acetyl-CoA and HeLa cell core histones [50].

### Fly transgenesis, polytene chromosomes and β-galactosidase assays

Maintenance of *Drosophila *cultures and generation of *P *transformant lines were done as previously described [51]. Male and female larvae were identified based on the size of the genital disc. *arm-Gal4*, UAS*-lacZ *and UAS-RedStinger lines were obtained from the Bloomington *Drosophila *stock center. Polytene chromosome squashes and immunostaining were carried out as described previously [52]. β-galactosidase assays were performed on hemisected adults as described previously [38]. Assays were performed in triplicate on 3 separate collections. The β-galactosidase activity was standardized by total protein microassays (Bio-Rad). Means and standard deviations of ratios were calculated from the 3 separate collections. Statistical analyses of β-galactosidase activities were performed using the mini-tab and SAS software packages.

### Chromatin Immunoprecipitation Assays

Chromatin immunoprecipitation assays using male and female third instar larvae were performed as described previously [53]. Undiluted immunoprecipitated DNA (2 μl) and 100-fold diluted input DNA (2 μl) were assayed with each primer set in triplicate. The primer pairs used were *Gpdhr *(5'-GTGCCCGACCTGGTTGAG-3") and *Gpdhf *(5'-CTTGCCTTCAGGTGACGC-3"), *armr *(5'-TTCCAAGACACAGAGAGGGTG-3") and *armf *(5'-GCCCTCGACAATCTCCTCC-3"), *pgd*10f (5'-GAAGGGCACGGGCAAGTG-3") and *pgd*10r (5'-CAATGCCGCCGTAATTAAGTCTC-3"), *lacZ*-2823F (5'-GCGCGAATTGAATTATGGCCC-3") and *lacZ*-2950R (5'-GCCATGTGCCTTCTTCCG-3"), *lacZ*-1540F (5'-GCTGTGCCGAAATGGTCC-3") and *lacZ*-1670R (5'-CGAAACGCCTGCCAGTATTTAG-3"), lacZ-324F (5'-GGTCAATCCGCCGTTTGTTC-3") and *lacZ*-493R (5'-TGTCCTGGCCGTAACCG-3").

Fold enrichment was determined by 2^(CP input *lacZ or control gene *- CP ChIP *lacZ or control gene*)/2^(CP input *Gpdh *- CP ChIP *Gpdh*).

Quantitative real-time PCR was conducted in triplicate using the LightCycler FastStart DNA MasterPLUS SYBR Green I reaction mix (Roche) in a LightCycler Instrument (Roche). An annealing temperature of 55°C and an extension time of 18 s were used.

The crossing point (CP) was automatically determined by the LightCycler software (Roche).

### RNA isolation, cDNA synthesis and Quantitative real-time PCR

UAS-DsRed.T4-NLS females were crossed to males with Gal4-MOF, Gal4-MOF mutant or Gal4[DB] transgenes. The 3^rd ^instar larval offspring were collected, sorted by sex and checked for red fluorescence by stereo fluorescence microscopy. Total RNA was extracted with TRIzol™ and treated with turbo DNase (Ambion). cDNA was synthesized using the SuperScript III First-Strand Synthesis SuperMix (invitrogen). RNA isolation and cDNA synthesis was carried out from three independent experiments. The sequences of DsRed primers are forward primer: 5'-GCGTGATGAACTTCGAGG-3' and reverse primer: 5'-GCCCATAGTCTTCTTCTGC-3". For normalization, we used primers for *pka *transcripts: forward primer: 5'-TTCTCGGAGCCGCACTCGCGCTTCTAC-3", reverse primer: 5'-CAATCAGCAGATTCTCCGGCT-3". qRT-PCR reactions were performed using Maxima™ SYBR Green/ROX qPCR Master mix (Fermentas), amplifications were run on standard 384-well reaction plate using Applied Biosystems7900HT Fast Real-Time PCR System. PCR efficiencies were determined from the slopes of standard curves from cDNA dilution series (triplicate). DsRed mRNA levels were calculated from the C_T _values of triplicate samples and three independent experiments using the 2^-ΔΔCT ^method.

## Authors' contributions

AHS, FL, VMW, KCK, SAM and MJS designed experiments. AHS, FL, VMW, EJB, KCK and MJS performed experiments. SAM compared hMOF and yEsa1 structures, designed active site mutants and prepared figures. MJS conceived of the study and wrote the manuscript with input from all authors. All authors read and approved the final manuscript.

## Supplementary Material

Additional file 1 **Figure S1. An X-linked UAS-RedStinger line is activated more strongly in females than males by Gal4-MOF**. Gal4-MOF line S41 was crossed with UAS-RedStinger3 ([FlyBase ID FBst0008545]) and the third instar larval offspring were examined for nuclear red fluorescence. Click here for file

Additional file 2 **Figure S2. H4K16ac is enriched at 3 × UAS*-arm-lacZ *and UAS*-lacZ *reporter genes in the presence of Gal4-MOF **Independent ChIP experiments were performed with nuclei isolated from larvae that carry *hsp83*-Gal4-MOF and either UAS-*lacZ or *3 × UAS*-arm-lacZ *transgenes. See main text for details Click here for file
