# Supplementary figures and images for: Sex-biased transcription enhancement by a 5' tethered Gal4-MOF histone acetyltransferase fusion protein in Drosophila
Source: BMC Mol Biol. 2010 Nov 9;11:80. doi: 10.1186/1471-2199-11-80 (PMC2988783; doi:10.1186/1471-2199-11-80)

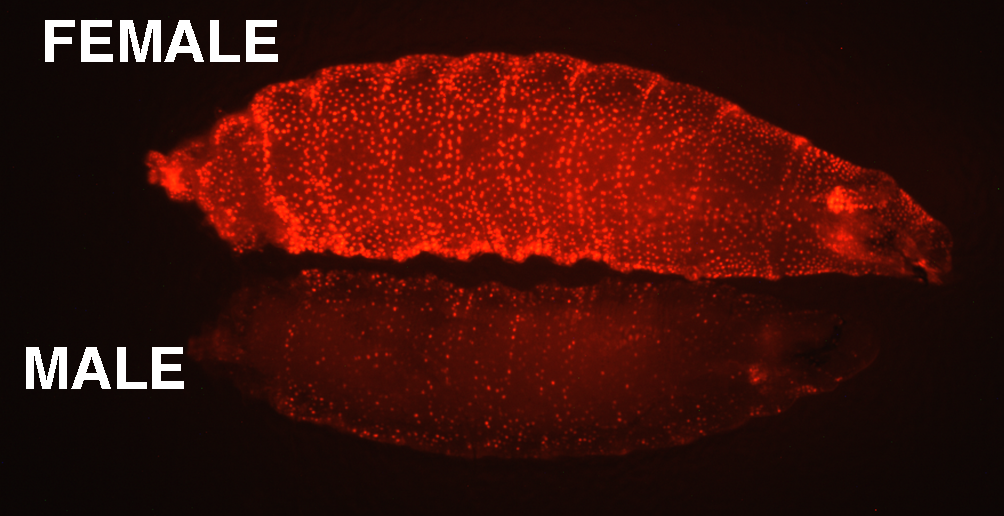

Supplement: Additional file 1 — Figure S1. An X-linked UAS-RedStinger line is activated more strongly in females than males by Gal4-MOF. Gal4-MOF line S41 was crossed with UAS-RedStinger3 ([FlyBase ID FBst0008545]) and the third instar larval offspring were examined for nuclear red fluorescence. [file 1471-2199-11-80-S1.PNG]

## H4K16ac Fold Enrichment

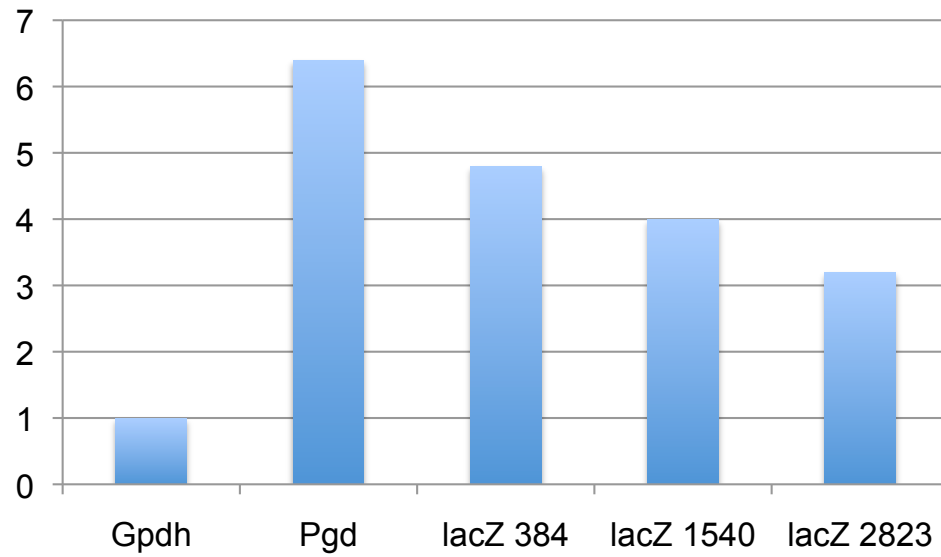

Gal4-MOF X UAS-lacZ

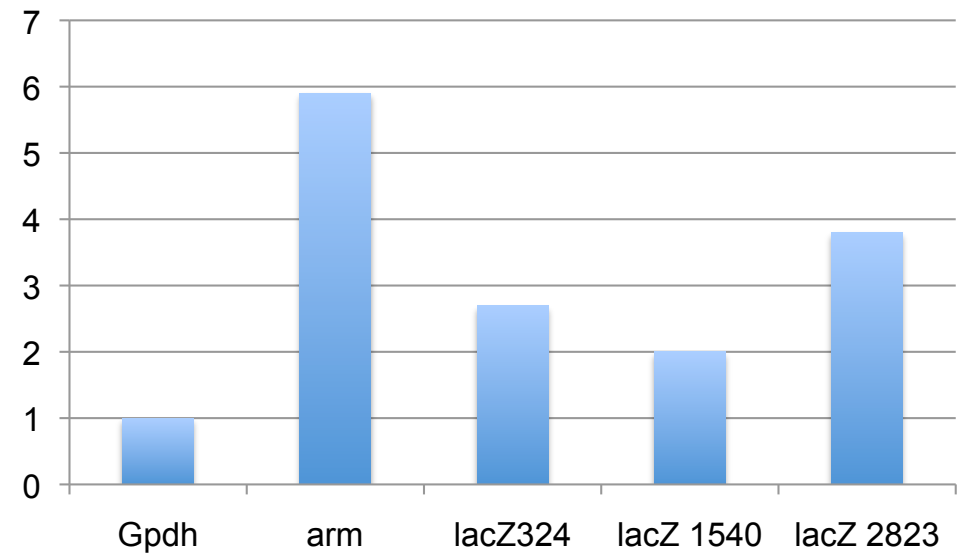

Gal4-MOF X 3xUAS-arm-lacZ

Supplement: Additional file 2 — Figure S2. H4K16ac is enriched at 3 × UAS-arm-lacZ and UAS-lacZ reporter genes in the presence of Gal4-MOF Independent ChIP experiments were performed with nuclei isolated from larvae that carry hsp83-Gal4-MOF and either UAS-lacZ or 3 × UAS-arm-lacZ transgenes. See main text for details [file 1471-2199-11-80-S2.PDF]
